# Heteronormative and cisnormative stereotypes in commonly-used social anxiety scales: A scoping review

**DOI:** 10.1016/j.xjmad.2024.100091

**Published:** 2024-10-11

**Authors:** Chantal Kasch, Cameron E. Lindsay, Stefan G. Hofmann

**Affiliations:** Philipps-University of Marburg, Department of Psychology, Schulstraße 12, D-35037 Marburg, Germany

**Keywords:** LGBTQIA+, Social anxiety scales, Heteronormativity

## Abstract

Social anxiety is a common, but under-researched topic in the LGBTQIA+ community. We conducted a scoping review of studies between January 2014 and April 2024 that included LGBTQIA+ samples with social anxiety scales evaluating scales for outdated language. The search produced 1155 results out of which 17 articles fit the inclusion criteria. The results suggest that 47 % of the measures include items that reinforce heteronormative or cisnormative stereotypes and 29.4 % of studies included scales inappropriate for the population under investigation. Therefore, the wordings of certain items need to be adapted to fit the investigated population and a re-examination of the psychometric properties of the revised scales is needed.

## 1 Introduction

Social anxiety is defined as a fear of social situations where there is a risk of embarrassment or negative evaluation, often involving distress about one’s role, status, and behavior [1]. Social anxiety is prevalent within the LGBTQIA+ communities that are at a higher risk due to social stigma and other minority stressors [2], [3], [4], [5], [6]. These minority stressors can differ based on sexual or gender minority status, but are generally differentiated between distal (from the environment) or proximal (from within) factors [3], [4], [7], [8]. Especially experiences of heterosexism and discrimination, internalized homonegativity, sexual identity concealment, rejection sensitivity, and sexual identity development have been found to be positively related to social anxiety in LGBTQIA+ populations [9].

There has been a call to update standard practices in relation to the research including the LGBTQIA+ community since as early as 2012; when Weiss et al. [10] published a paper discussing the heterocentric nature of social anxiety scales, as well as providing alternative wordings for many of these scales. Weiss et al. [10] further highlight that heterocentric language can lead to a non-response bias. The current scoping review examines sex and gender stereotypes found in social anxiety scales in LGBTQIA+ studies published in the last decade (between 2014–2024).

The term “opposite sex” is found throughout many social anxiety scales and has been critiqued often with several attempts to correct the problematic language [10], [11]. As noted by Weiss [10], the term “opposite sex” in social anxiety scales has three main problems. The first is how confusing the term “opposite sex” makes a question for both queer and heterosexual participants, as ‘interactions with the opposite sex’ can be broadly perceived as romantically or platonically. Moreover, in the romantic perception, lesbian, bisexual, and gay participants are excluded from the question. The second point refers to how many of these scales were developed with heterosexual participants in mind, rather than anyone residing outside of the heteronormative. The third discusses how the terminology of “opposite sex” alienates queer participants, damages rapport, and decreases compliance [10].

Sex, generally seen as a binary construct between ‘male’ and ‘female’, expands beyond the archetype of ‘sex equating to genitalia,’ as the terminology ‘sex’ refers to biological underpinning not simply genitalia [12], [13]. Therefore, the term can also include bodily features, or other physical characteristics like chromosomes or secondary anatomy [12], [13]. Some countries now allow for intersex or non-binary classification instead of the widely used binary “male” and “female” [14]. Gender, on the other hand, is formulated by society's view of bodily and perceptual differences and is usually defined with a focus on the social construct of relationship roles, behaviors, and socially assigned stereotypes [12], [13], [15]. The variability of these terms may seem colloquially equal, however, in the field of psychology it is monumentally different. Notably, however, there are cross-cultural differences in what is considered sex and gender. Western cultures mostly promote a binary gender concept with clear lines between what is understood to be “a woman” and “a man”. The assumed default of normative society includes cisnormativity, the assumption that gender is confined to the binary of male and female. Combined with heteronormativity, the assumption that all people are by default straight, it creates social constructs excluding everyone who does not inherently adhere to this “norm” [15]. Gender identity can vary from person to person and within the person. Examples of gender identity terms are cisgender, nonbinary, agender, or genderqueer. The term non-binary describes an identity, where a person may identify with both “man” and “woman” or neither. It can also be used as an umbrella term [16], [17]. Genderqueer further includes an idea of fluidity of gender identity [16]. Agender could describe a person’s lack of gender identity or be seen as a gender identity in itself [15].

Non-Western cultures on the other hand have been fostering more diverse environments including gender identities in a nonbinary understanding [18]. Recently there has been a shift in Western societies to expand the definition of gender as a construct which includes identities outside of a cisnormative understanding. Gender identity refers to one sense of self, which means the personal understanding of one’s gender may or may not correspond to the sex assigned at birth [15]. It should not be assumed that a person’s gender identity is congruent with how they present.

Sexual orientation, on the other hand, solely refers to romantic, emotional, or sexual attraction (or lack thereof) to other people [15], common terms used to describe sexual orientation are lesbian, gay, bisexual, straight, or asexual. Popular acronyms used to refer to the communities are LGBTQIA+ , which stands for lesbian, gay, bisexual, transgender, queer, Intersex, Asexual, the plus including any other identity not named (umbrella term, versions may differ based on location) [15]. A newer acronym gaining popularity is SOGIE (Sexual Orientation, Gender Identity and Expression) [15]. It is to note that this list is not comprehensive and was drawn from a Western perspective in 2024, descriptions and terms may differ in other countries and cultures. In an attempt to use the most inclusive language available the term queer is used as an umbrella term in this paper to include all people and identities within the LGBTQIA+ community, as the word has been reclaimed by wide parts of the community [16]. It is important to note that a person can identify as queer as their sexual orientation or gender identity or use it as an umbrella term [16], [17].

The recent literature [12], [13], [18], [19], [20], [21] highlights the ongoing need to educate oneself about language used within and outside of the communities researched, as well as how important it is to refrain from language that might be stigmatizing or making assumptions about participants, further underlining the fact that all research should be affirming, not just research investigating queer samples [19].

Griffith et al. [19] explains that certain terminology is outdated and inconsistent with APA Guidelines that were published as a framework on how mental health professionals and researchers should work with sexual minority and transgender-nonconforming persons [22], [23]. Along that same thread, it is standard ethical practice to review all items and language used in questionnaires and it should be explained in the method section of a paper if questions have been modified to fit the target population or be highlighted as a limitation if no adjustments were made to problematic items [19]. This will add to a better understanding for the reader, accurate replication and moreover effective science communication.

In the past, the terms “sex” and “gender” were often used interchangeably. Today, there are clear boundaries between the two words [24]. Modern linguistics details how “sex” refers to biology (see Table 1), however many social anxiety scales (see table 3) refer to ‘attraction to the opposite sex’ thus making the prompt unclear if the attraction is to the person or their biological characteristics [10], [12]. Either way this terminology negates many identities found in the LGBTQIA+ community [10]. Research shows that wordings in questionnaires can relay an underlying message about outdated stereotypes [25], [26], [27]. Upon further examination of research focused on social anxiety, particularly within queer samples and the LGBTQIA+ community, it is concerning to note the prevalence of studies that employ rating scales containing wording that reinforces heteronormativity (see Table 1) [10]. Additionally, heteronormativity or cisnormativity furthers the creation of stigma and microaggressions. Expressions of microaggression include verbal or nonverbal, behavioral, or environmental actions which communicate derogatory attitudes; while being conscious or unconscious and perpetrators may be unaware of their communication evoking feelings of exclusion, invalidation, and inferiority [28]. Some of the most common sexual and gender identity microaggressions include: discomfort or disapproval of the LGBTQ experience, use of heterosexist or transphobic terminology, microaggressions as humor, endorsement of heteronormative/gender conforming culture or behavior, assumption of universal experiences, and assumptions of sexual pathology or abnormality [29].

**Table 1:** Database search result.

An example of purposefully reevaluating scales is an item of the Social Interaction Anxiety Scale, originally created by Mattick and Clarke [30], as item 14 in the original scale reads as “I have difficulty talking to attractive persons of the opposite sex”. The use of “opposite sex” in the relation to attraction is quite problematic with a sexual minority sample as it does not reflect their sexual orientation. Lindner et al. [11] validated a non-heteronormative version of the Social Interaction Anxiety Scale [30]. Yet, the frequency of utilizing the reassessed scale and the prevalence of opting for inappropriate scales over adapted ones in LGBTQIA+ research remain open questions.

Based on the previous literature and overall trends within LGBTQIA+ research, we hypothesized that several of the frequently used social anxiety scales may not be conducive towards modern society. Additionally, we hypothesize that throughout LGBTQIA+ research there may be a large amount of the outdated scales used without adaptation to fit the community.

## 2 Methods

For this scoping review of the most commonly used social anxiety scales in LGBTQIA+ research, we restricted our search to 2014 to 2024 to focus on literature during the last decade. We adopted the PRISMA extension for scoping reviews [31] to examine the commonly used scales for use of heteronormative/cisnormative language, as well as if the scales include references to sexual contexts (i.e. Trying to make someone's acquaintance for the purpose of a romantic/sexual relationship; LSAS [32]). We followed the Joanna Briggs Institute Manual [[33], see p. 413] for transparency on analysis methods. Including full details on inclusion/exclusion criteria, search strategy, rationale, and procedures [34]. Additionally, this study was pre-registered to the Open Science Framework (https://osf.io/s2vq5/?view_only=10efc340441c40d0813d30c389df7aa6).

### 2.1 Publication Selection

We searched Psycinfo, Psycarticles, Pubmed, Science Direct, Google Scholar, Springerlink, and SAGE Journals for studies published between January 2014 and April 2024 using the search term “LGBTQIA+ ” AND “Social Anxiety.” Articles were categorized based on which social anxiety scale they used and the population being studied. The search terms also produced literature using terms as “LGB” or “sexual minority” or “gender minority”.

### 2.2 Eligibility criteria

Studies were included if they (a) included a social anxiety scale and had a LGBTQIA+ sample or subsample, (b) conducted with participants above the age of 18, (c) were of academic integrity with empirical foundations, meaning studies included were peer reviewed and considered a “research article.” Articles were excluded if they were non-empirical book chapters, meta-analyses, systematic reviews, narrative reviews, qualitative studies, did not include a social anxiety scale, did not include a LGBTQIA+ sample, included participants under 18 years old, were clinical or diagnostic, and if articles were generally unrelated to all identified constructs. No language restrictions were applied.

### 2.3 Search and screening process

Out of 1155 records, 17 papers were identified that fit the inclusion criteria. Screening was completed by two independent raters and then were compared and placed into one comprehensive analysis. Articles were first screened by abstract for possible fit of the inclusion criteria before being screened fully. Conflicts were dissolved by discussion between the two raters after the initial screening and consulting with a third independent rater, if necessary (Table 1). The raters used Rayyan, an online screening application, to review all articles [35].

## 3 Results

A total of 17 studies met the inclusion criteria, the most commonly used social anxiety scale was the Social Interaction Anxiety Scale including a validated version and a version using just the straightforward items [30], [36], [37], being used four times across LGBTQIA+ samples. The second most commonly used scales were the Liebowitz Social Anxiety Scale [LSAS; 32], and the mini version of the Social Phobia Inventory [Mini-SPIN; 37], each being used in three different studies across the LGBTQIA+ community. Other commonly used scales, used two times, were the Social Phobia Inventory [SPIN; 38], and adapted versions of the SIAS (one study adapted question 14 individually, the other used a validated adapted version of the SIAS [11]). These studies evaluated sexual and gender minorities, including a non-identified LGBTQIA+ subgroup as men who have sex with men. There were five scales, across the LGBTQIA+ spectrum, which were used once each, including the Brief Fear of Negative Evaluation [39], the State Social Anxiety Questionnaire [40], the revised version of the Brief Fear of Negative Evaluation [41], the self-report version of the LSAS [42], and the Symptoms-Checklist-27-Plus [43].

Next, we analyzed the most commonly-used social anxiety scales, as well as other well-known social anxiety scales for heteronormative and cisnormative language and sexual contexts. Amongst commonly used social anxiety scales 5 include heteronormative language, cisnormative language, or sexual contexts. Further, out of the 17 included studies, six used scales with heteronormative, cisnormative, or sexual context language. Moreover, 29.4 % of all included scales were used in contradictory populations (e.g. the use of questions including “opposite sex” were used in studies for lesbian, gay, or bisexual samples) (Table 2).

**Table 2:** Commonly-used social anxiety scales.

Scale	Heteronormative, cisnormative language	Sexual context	Number of items (out of total) with problematic language	Number of times scale was used in Queer samples	Samples
LSAS [32]	No Heteronormative, Cisnormative Language	21. Trying to make someone's acquaintance for the purpose of a romantic/sexual relationship	1/21	3	Trans, Transgender/ Sexual Minorities/ Gay, Bisexual Men
SPIN [38],	No Heteronormative, Cisnormative Language	No Sexual Contexts	0/14	2	Sexual Minority Men, Men who have Sex with Men
Mini-SPIN [37]	No Heteronormative, Cisnormative Language	No Sexual Contexts	0/3	3	Trans/Gender Non-conforming/Sexual Minority Men
BFNE [39]	No Heteronormative, Cisnormative Language	No Sexual Contexts	0/12	1	Sexual Minority
BFNE-R [41]	No Heteronormative, Cisnormative Language	No Sexual Contexts	0/12	1	Sexual Minority Adults
Updated SIAS [11], Adapted SIAS in Study	No Heteronormative, Cisnormative Language	No Sexual Contexts	0/20	2	Gay, Bisexual Men/Sexual Minorities/ Gender Minorities
State Social Anxiety Questionnaire [40]	No Heteronormative, Cisnormative Language	No Sexual Contexts	0/7	1	Lesbian
Symptoms- Checklist−27-Plus [43]	No Heteronormative, Cisnormative Language	No Sexual Contexts	0/27	1	Gay
SIAS [30]	14. I have difficulty talking to attractive persons of the opposite sex	No Sexual Contexts	1/20	3	Gay, Bisexual Men

Additional Commonly-Known Social Anxiety Scales for Consideration					

SAQ-A30 [44]	4. Asking someone attractive of the opposite sex for a date	21. Being asked out by a person I am attracted to	6/32	0	
	6. Feeling watched by people of the opposite sex	29. Asking someone I find attractive to dance			
	24. Starting a conversation with someone of the opposite sex that I like	32. Telling someone I am attracted to that I would like to get to know them better			
SADS [45]	7. I am usually at ease when talking to someone of the opposite sex	No Sexual Contexts	2/28	0	
	10. I often feel nervous or tense in casual get-togethers in which both sexes are present	No Sexual Contexts			
SPAI [46]	"Opposite Sex" and more	No Sexual Contexts		0	

Note: LSAS = Liebowitz Social Anxiety Scale [28], SPIN = Social Phobia Inventory [34], BFNE = Brief Fear of Negative Evaluation [35], BFNE-R = Brief Fear of Negative Evaluation Revised [37], SIAS = Social Interaction Anxiety Scale [11], [26], SAQ-A30 = Social Anxiety Questionnaire - Adults [40], SADS= Social Anxiety Distress Scale [41], SPAI = Social Phobia and Anxiety Inventory [42].

## 4 Discussion

We surveyed the literature for heteronormative and cisnormative stereotypes appearing in commonly used social anxiety scales within the last decade. Although there have been many historic cultural-adaptations to the LGBTQIA+ community since the publication of the study by Weiss et al. [10], the results of our scoping review identified multiple instances for inappropriate use of the scale in queer samples. We found that many of the surveyed scales still use heteronormative language, cisnormative language or include sexual contexts which are not suitable for all members of the LGBTQIA+ community. Specifically, we found 2 of the social anxiety scales used within 6 studies investigating queer samples included one or more heteronormative or cisnormative items or sexual contexts, and 29.4 % of scales were unfitting for the use with the study samples (i.e. using “opposite sex” in a sample of gay men).

Choosing scales solely based on popularity or convenience is associated with a risk for bias on multiple levels, especially in studies that are designed to investigate queer samples [18]. One such risk for bias is in the validity of the study itself. With questions that are not inclusive to the participants, the psychometric properties of the scale become questionable. In their review, Weiss et al. [10] found participants did not respond to questions that were not appropriate or applicable to them, resulting in a non-response bias. This non-response bias would then become vitally important to consider in analyses as it may change the overall results of the study.

Moreover, the inclusion of questions which are biased to fit the binary, non-binary participants could feel invalidated and harmed, leading to microaggression. A common thread interweaving amongst most qualitative studies evaluating sexual and gender minority microaggressions was expression and maintenance of heteronormativity [47], [48]. A recent study evaluating sexual and gender identity microaggressions in sexual and gender minority youth found more than half of the participants experienced heterosexist or transphobic terminology microaggressions [29]. In 2022, in a qualitative study with sexual and gender minority patients in a healthcare setting, 177 participants encountered cisnormative and heteronormative language and sexual orientation or gender assumptions, which made up 85.3 % of the encounters [49]. These studies show how using heteronormative language can be seen as a way to foment the queer community through heterosexist microaggressions. Additionally, using scales with heteronormative language within general population samples poses another problem of exclusion. Upkeeping heteronormative standards could be perceived as stigmatizing and non-welcoming to queer participants. This not only makes it harder to reach queer individuals to take part in research but could create further biases.

We evaluated the continued use of heteronormative social anxiety scales in queer samples, highlighting the quintessential need to reevaluate research methodologies to ensure researchers are not expressing heteronormative microaggressions. Relying on diversity training for therapists and clinicians is not enough [50]. To ensure inclusivity and representation for everyone interested and involved in research, we recommend, in line with the latest guidelines and reviews [18], [19], [22], [23], to establish that external and internal validity in scales are upheld. Specifically, we suggest that when investigating social anxiety in queer samples, scales with neutral language or scales developed for use in the LGBTQIA+ community should be prioritized; when investigating a general sample, scales with neutral items should be picked as to not assume the identity of study participants; researchers should be aware whether the scales they include reflect the population and era they are working in. We recommend the SPIN, BFNE or the State Social Anxiety Questionnaire as these questionnaires use the most inclusive language. If instruments exist in different versions, we recommend that investigators carefully examine in items for heteronormative and cisnormative stereotypes.

Overall, our study found there have been improvements made regarding the use of non-heteronormative and non-cisnormative social anxiety scales. However, there is still further improvement which needs to occur, as many of the studies evaluating social anxiety in queer samples over the last decade used scales with heteronormative language, cisnormative language, or sexual contexts. The continual growth and mindfulness of the scientific community is imperative to ensuring the constituents we serve are not harmed by our practices. We suggest adapting scales to be more inclusive as the first step towards creating an inclusive and equitable platform where reliable, valid, and ethical research is conducted. The literature shows that allowing participants to self-identify, especially within demographic sections in psychiatric materials, is beneficial in creating inclusive spaces [51]. In addition to normalizing neutral language in scales, it is especially important to consider wording in demographic items and when formulating or choosing questions about romantic interest. For example, replacing “husband/wife” with “partner” or “significant other” or using “persons of romantic interest” instead of “person of the opposite sex” in items specifically referring to romantic relationships, rids the items of bias. It is not necessary to specify who someone is attracted to or what that person's gender identity might be to assess whether the participant is afraid of interactions, however it can be beneficial to add appropriate demographic items to ensure proper recording of the sample [12], [21].

The use of inclusive language will aid in the deconstruction of cisnormativity and heteronormativity. It will also allow queer participants to feel welcome in all areas of research, not just in those studies directly pertaining to queer individuals, as it reduces the pressure of having to identify with the predetermined terms or labels. Additionally, if implemented thoroughly, it will make it easier to compare and generalize results across cultures and populations.

When constructing scales and questionnaires, researchers should ask themselves whether the wording of items makes assumptions about the identity of possible participants. Additionally, we suggest that researchers pay close attention to used wording and a possible need for changes or revalidations.

The main limitation of the study is that social anxiety is not well studied in queer samples. With a small number of studies to include it is difficult to fully understand the impact of heteronormative and cisnormative language imposing on the queer community. Future research should consider studying the impact the use of these scales has on the queer community.

## Declaration of Competing Interest

The authors declare that they have no known competing financial interests or personal relationships that could have appeared to influence the work reported in this paper.
